# Supplementary figures and images for: Great Diversity of Bacterial Microbiota in Thai Local Food: “Tai-Pla”, the Salty Fermented Fish-Entrail Sauce
Source: Foods. 2025 Nov 29;14(23):4104. doi: 10.3390/foods14234104 (PMC12692573; doi:10.3390/foods14234104)

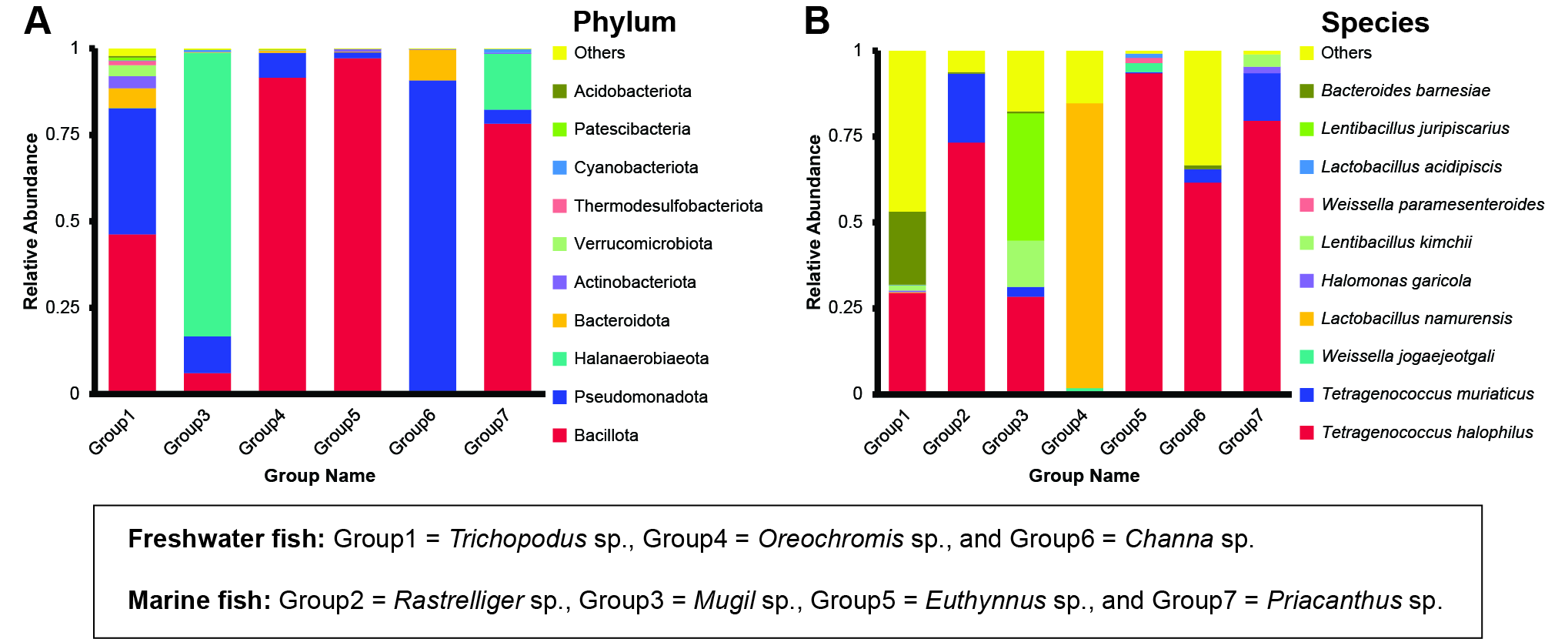

Supplement: Supplementary file 1 [file foods-14-04104-s001.zip › Figure S1.tif]

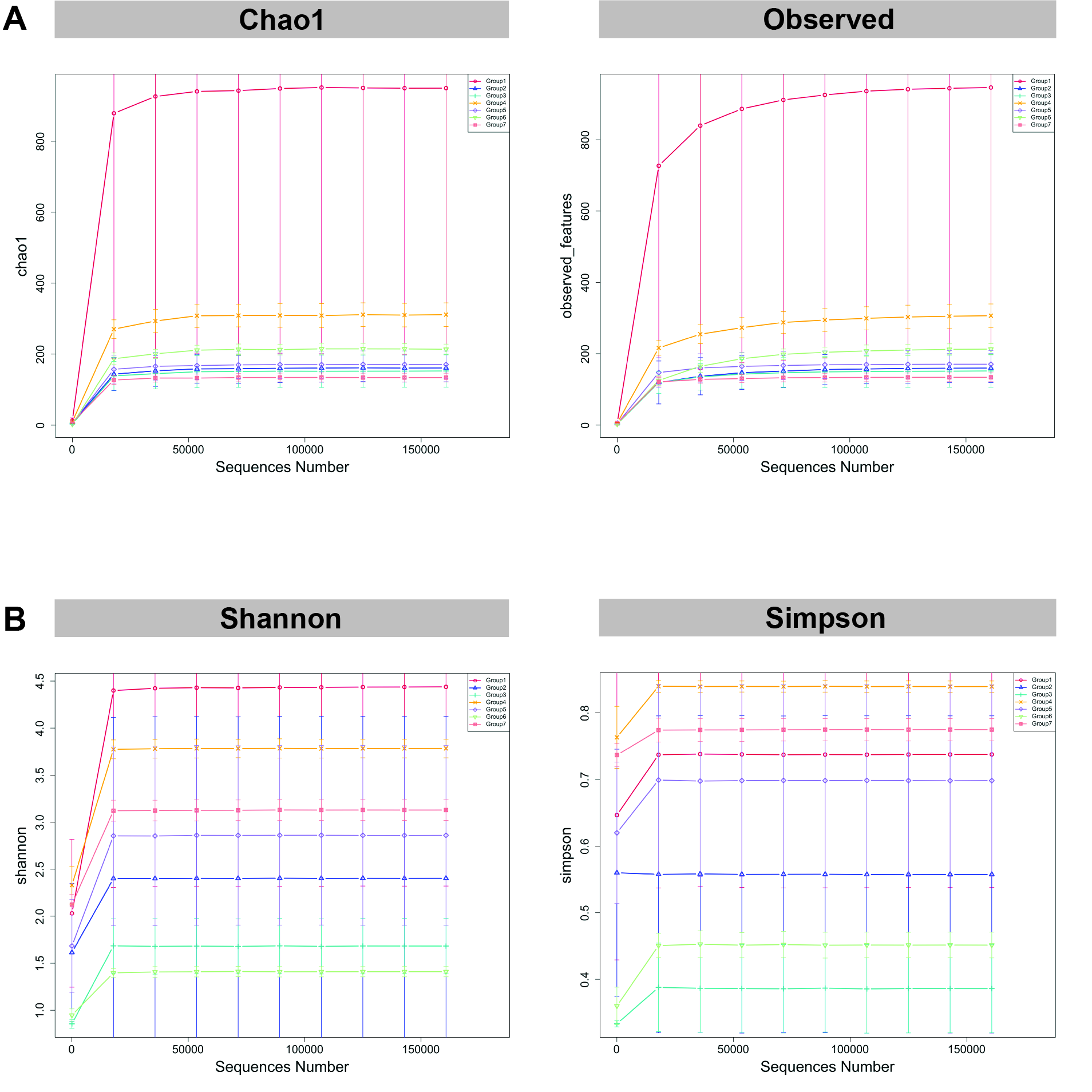

Supplement: Supplementary file 1 [file foods-14-04104-s001.zip › Figure S2.tif]
